# PD-1 and PD-L1 inhibitors after platinum-based chemotherapy or in first-line therapy in cisplatin-ineligible patients: Dramatic improvement of prognosis and overall survival after decades of hopelessness in patients with metastatic urothelial cancer

**DOI:** 10.1007/s12254-018-0396-y

**Published:** 2018-03-08

**Authors:** Irene Resch, Shahrokh F. Shariat, Kilian M. Gust

**Affiliations:** 1 Department of Urology, Comprehensive Cancer Center, Vienna General Hospital, Medical University of Vienna, Währinger Gürtel 18–20, 1090 Vienna, Austria; 2 Karl Landsteiner Institute of Urology and Andrology, Vienna, Austria; 3 Department of Urology, University of Texas Southwestern, Medical Center, Dallas, TX USA; 4 Department of Urology, Weill Cornell Medical College, New York, NY USA

**Keywords:** Checkpoint inhibitor, Immune checkpoint, Advanced urothelial cancer, Metastatic urothelial cancer, Cisplatin ineligible, Biomarkers

## Abstract

Until recently, there were no true innovations in the management of locally advanced (aUC) and metastatic urothelial cancer (mUC) in the last three decades. Vinflunine has been approved by the EMA (European Medicines Agency) with only limited improvement compared to best supportive care in second line treatment. In addition, gemcitabine/cisplatin has been established as an alternative to methotrexate, vinblastine, doxorubicin, and cisplatin (MVAC). The advent of checkpoint inhibitors (CPI) revolutionized the care of these patients, transforming a unanimously deadly disease into one with hope through sustained disease control. Five immune CPI have recently been approved for aUC/mUC by the US Food and Drug Administration (FDA) including atezolizumab, nivolumab, pembrolizumab, durvalumab and avelumab. All five CPI are FDA-approved as second-line therapy with atezolizumab and pembrolizumab also being approved for first-line therapy in cisplatin-ineligible patients. The rapid acceptance in the treatment algorithm of UC is based on the impressive clinical efficacy of these agents in some patients, combined with their excellent safety profile. These new agents are indeed the most important advancement in UC care. However, the challenge in the age of precision medicine is to identify the patients who are most likely to benefit from CPIs, as the majority of patients do not respond to CPI. Toward this goal, validation of clinical, molecular and imaging biomarkers that serve for prediction and monitoring of treatment response are of central necessity.

## Introduction

UC (urothelial cancer) is a common malignancy with aggressive tumor biology so that patients with locally advanced or metastatic disease stage have a dire prognosis with almost unanimous mortality. Cytotoxic platinum-based chemotherapy is the current standard treatment for patients with locally advanced (aUC) or metastatic UC (mUC). However, cisplatin is associated with significant toxicities; therefore, about 50% of patients are ineligible to receive cisplatin-based cytotoxic therapy [1].

In 2016, the United States (US) Food and Drug Administration (FDA) approved atezolizumab, followed by nivolumab, durvalumab, avelumab, and pembrolizumab for the treatment of patients with locally advanced and mUC in the post-platinum setting. In 2017, atezolizumab and pembrolizumab also received FDA approval for cisplatin-ineligible patients in the first-line setting. This class of agents have awakened new hope in this forgotten disease.

## CPI in second-line after platinum-based chemotherapy

The second-line treatment of mUC has changed dramatically with the FDA approval of checkpoint inhibitors (CPIs). Until 2016, vinflunine was the only approved chemotherapeutic agent that showed an improved survival in platinum-refractory patients in a phase III trial with a benefit in median overall survival (OS) of 2.3 months compared to best supportive care [2]. This made it the treatment of choice in 2nd line mUC in Europe despite its non-negligible toxicity. In the US, taxanes and gemcitabine were the most commonly used chemotherapeutic agents.

The monoclonal antibody against programmed cell death-1 (PD-1), pembrolizumab, showed antitumor activity in patients with mUC after disease progression or recurrence following platinum-based chemotherapy. The phase III KEYNOTE-045 trial compared pembrolizumab to chemotherapy of investigator’s choice (paclitaxel, docetaxel or vinflunine) after disease progression or recurrence following platinum-based chemotherapy. Pembrolizumab had an improved median OS of 10.3 (95% CI 8.0–11.8) compared to 7.4 months for chemotherapy (95% CI 6.1–8.3). This was independent of the programmed cell death ligand‑1 (PD-L1) status of the tumor. The objective response rate (ORR) was 21.1% for pembrolizumab compared to 11.0% for chemotherapy (*p* = 0.002). The estimated OS rate at 12 months was 43.9% (95% CI 37.8–49.9) for pembrolizumab, as compared to 30.7% (95% CI 25.0–36.7) for chemotherapy. Patients treated with chemotherapy experienced more general and high grade (grade 3 and 4) AEs than those who received pembrolizumab (all AEs 90.2% vs. 60.9%; with grade 3–5 AEs in 49.4 vs. 15.0%). Based on these findings, the FDA approved pembrolizumab as a second-line treatment for aUC and mUC [3].

The monoclonal PD-L1 targeting antibody, atezolizumab, was the first CPI to be FDA approved in May 2016 for mUC after failure of a platinum-based chemotherapy. The approval is independent of the PD-L1 status. Cohort 2 of the phase II IMvigor 210 trial included patients with disease progression during or following prior platinum-containing chemotherapy. Median OS was 11.4 months in patients with higher PD-L1 expression (IC2/3) and 7.9 months (95% CI 6.6–9.3) in the entire cohort. The majority of responders to treatment with atezolizumab (84%) had ongoing responses at a median follow-up of 11.7 months. Atezolizumab has a good safety profile with only 16% grade 3–4 adverse events (AE). The most common AE (any grade) were fatigue (30%), nausea (14%) and decreased appetite (12%) [4–6]. IMvigor 211, a randomized phase III trial comparing atezolizumab to chemotherapy in the second line setting failed to prove a significant benefit of atezolizumab over chemotherapy of investigator’s choice (paclitaxel, docetaxel or vinflunine) in the primary endpoint OS, which did not show a significant difference in PD-L1 high expressing patients and therefore did not allow any further analysis due to a prespecified hierarchical statistical analysis [7].

Nivolumab, a monoclonal PD-1 antibody has been evaluated in CheckMate 275, a phase II, open-label, single-arm, multicenter study. Results led to an accelerated FDA approval in February 2017 for patients with aUC or mUC with disease progression during or following platinum-based chemotherapy. The median OS for nivolumab was 8.74 months with ORR of 19.6% and a median (progression free survival) PFS of 2 months. Complete response (CRR) was observed in 2%, partial response in 17%, and stable disease was documented in 23% of patients. Median time to response was 1.9 months. All together 64% of patients showed immune related AE of any grade, most frequently seen were fatigue (17%), pruritus (9.3%) and diarrhea (8.9%). However, clinically relevant grade 3 or 4 treatment-related AEs occurred in only 18% and treatment discontinuation due to AE in only 5% of patients [8].

Durvalumab is a human monoclonal antibody targeting PD-L1. In May 2017, the FDA granted accelerated approval of durvalumab for aUC/mUC based on the results from the single-arm phase I/II trial 1108. This trial included patients with disease progression after platinum-based chemotherapy [9]. The ORR was dependent on PD-L1 expression, 31.0% (95% CI 17.6–47.1%) in the entire cohort and 46.0% (27.5–66.1) in patients with positive PD-L1 score (≥25% expression on either tumor or immune cells). Patients a negative PD-L1 score (<25% on tumor and immune cells) did not respond to treatment with durvalumab. In 23% of cases immune-related AEs were documented, most common were fatigue, musculoskeletal pain, constipation, nausea, decreased appetite, peripheral edema, and urinary tract infection. However, it is notable that 43% of patients showed serious AEs (grade 3 or 4) [9].

The PD-L1 targeting antibody avelumab has been FDA approved for the treatment of aUC/mUC following a platinum-based chemotherapy. In the phase Ib trial JAVELIN, safety and efficacy of avelumab had been evaluated. The ORR was 18.2% and median OS was 13.7 months for the entire cohort, while it is notable that patients positively tested for PD-L1 expression (5% or more tumor cells expressing PD-L1) showed a higher ORR of 53.8%, while only 4.2% of PD-L1 negative patients responded to treatment with avelumab 4.2%. In the entire cohort, partial responses were observed in 18/161 (11%) patients and 9/161 (6%) patients achieved a complete response. Infusion-related reactions (29%) and fatigue (16%) were the most frequent AE, while grade 3 or 4 AEs occurred in only 8% of patients [10, 11].

## CPI in first-line in cisplatin-ineligible patients

More half of patients with aUC and mUC are ineligible for cisplatin-based chemotherapy, primarily due to poor performance status, age, cardiovascular comorbidities and impaired renal function [1, 12]. Pembrolizumab and atezolizumab are approved for first-line therapy in cisplatin-ineligible patients with aUC or mUC [13, 14].

The recently published phase II trial KEYNOTE-052 evaluated pembrolizumab as first-line therapy for cisplatin-ineligible patients. Cisplatin-ineligibility was defined as presence of at least one of the following characteristics: ECOG performance status 2, creatinine clearance 30–60 ml/min, grade ≥2 audiometric hearing losses, peripheral neuropathy or NYHA class III heart failure. A confirmed ORR was defined as primary endpoint. In a median follow-up of 5 months, 370 patients with a median age of 74 years received pembrolizumab as first-line therapy. The ORR was 29% (95% CI 24–34) and CRR was 6%. At time of data cut off in 83% of 84 responders to treatment with pembrolizumab hand ongoing responses, while 78% of responders had a duration of response (DOR) of more than 6 months based on Kaplan–Meier estimates. Median time to response was 2 months and median DOR had not yet been reached at time of publication. Treatment success was associated with PD-L1 expression: 38% of patients with a combined positive PD-L1 score of 10% or more had an objective response. In patients with lymph node only disease the ORR was higher than in patients with visceral metastasis (40 vs. 21%). Despite an aged study population, 29% of patients were 80 years or older and presence of a poor performance status in 42% (ECOG 2), pembrolizumab had an acceptable tolerability and favorable safety profile and was granted accelerated approval for first line treatment of cisplatin-ineligible patients. Drug-related AEs of any grade (mostly fatigue) were experienced by 67% of patients. The most common grade 3/4 treatment-related AE were fatigue (2%), alkaline phosphatase increase (1%), colitis (1%), and muscle weakness (1%) and 10% of patients had serious treatment-related AEs.

Atezolizumab received accelerated FDA approval in April 2017 for the first-line treatment of patients with aUC/mUC who are ineligible for cisplatin-based chemotherapy based on the cohort 1 of the single arm phase II IMvigor210 trial. In this trial, the majority of the 119 cisplatin-ineligible patients included were cisplatin ineligible due to impaired renal function (71%). Primary endpoint was ORR and secondary outcomes were PFS and OS. With median follow-up of 17.2 months, the ORR was 23% (95% CI 16–31) with a CRR of 9%. The median PFS was 2.7 months (95% CI 2.1–4.2) and the median OS was 15.9 months (95% CI 10.4 to not estimable). The most common documented drug-related grade 3/4 AEs were fatigue (8%), anemia (7%), urinary tract infections (5%) diarrhea (5%), increase in the level of creatinine in the blood (5%), increase of the liver enzyme alanine transaminase (4%), hyponatremia (15%), decreased appetite (3%), and back and neck pain (3%) [14].

## Biomarkers

However, the challenge in the age of precision medicine is to identify the patients who are most likely to benefit from CPIs, as the majority of patients do not respond to CPI. Toward this goal, validation of clinical, molecular and imaging biomarker that serve for prediction and monitoring of treatment response are of central necessity.

The rapid implementation of PD-1/PD-L1 CPIs in the treatment algorithm of UC has led to a demand for predictive biomarkers. Being able to predict patients who are likely to benefit from CPI treatment could lead use of CPI in earlier treatment phases in patients with UC. To date, no reliable predictive biomarkers have been identified in UC. PD-L1 expression, which has been evaluated in all major trials, has failed to provide a reliable predictive value since patients with low or no PD-L1 also respond to treatment [4, 8, 9, 15]. In fact, PD-L1 expression had rather a prognostic value in the KEYNOTE-045 trial [3], where a higher expression of PD-L1 was associated with a worse OS in both groups. Further predictive biomarkers are currently under investigation, yet have not led to clinically useful results so far [16].
